# Re-Assembled Botulinum Neurotoxin Inhibits CNS Functions without Systemic Toxicity 

**DOI:** 10.3390/toxins3040345

**Published:** 2011-03-24

**Authors:** Enrico Ferrari, Elizabeth S. Maywood, Laura Restani, Matteo Caleo, Marco Pirazzini, Ornella Rossetto, Michael H. Hastings, Dhevahi Niranjan, Giampietro Schiavo, Bazbek Davletov

**Affiliations:** 1 Medical Research Council Laboratory of Molecular Biology, Cambridge CB2 0QH, UK; Email: eferrari@mrc-lmb.cam.ac.uk (E.F.); emaywood@mrc-lmb.cam.ac.uk (E.S.M.); mha@mrc-lmb.cam.ac.uk (M.H.H.); niranjan@mrc-lmb.cam.ac.uk (D.N.); 2 Istituto di Neuroscienze, Consiglio Nazionale delle Ricerche, 56100 Pisa, Italy; Email: restani@in.cnr.it (L.R.); caleo@in.cnr.it (M.C.); 3 Dipartimento di Scienze Biomediche, Università di Padova, 35121 Padova, Italy; Email: marco.pirazzini@studenti.unipd.it (M.P.); ornella.rossetto@unipd.it (O.R.); 4 Molecular NeuroPathoBiology Laboratory, Cancer Research UK London Research Institute, London WC2A 3LY, UK; Email: giampietro.schiavo@cancer.org.uk

**Keywords:** botulinum neurotoxin, nervous system, protein engineering, synapse, SNAREs, BOTOX, BITOX

## Abstract

The therapeutic potential of botulinum neurotoxin type A (BoNT/A) has recently been widely recognized. BoNT/A acts to silence synaptic transmission via specific proteolytic cleavage of an essential neuronal protein, SNAP25. The advantages of BoNT/A-mediated synaptic silencing include very long duration, high potency and localized action. However, there is a fear of possible side-effects of BoNT/A due to its diffusible nature which may lead to neuromuscular blockade away from the injection site. We recently developed a “protein-stapling” technology which allows re-assembly of BoNT/A from two separate fragments. This technology allowed, for the first time, safe production of this popular neuronal silencing agent. Here we evaluated the re-assembled toxin in several CNS assays and assessed its systemic effects in an animal model. Our results show that the re-assembled toxin is potent in inhibiting CNS function at 1 nM concentration but surprisingly does not exhibit systemic toxicity after intraperitoneal injection even at 200 ng/kg dose. This shows that the re-assembled toxin represents a uniquely safe tool for neuroscience research and future medical applications.

## 1. Introduction

The study of the nervous system of vertebrates, and ultimately human patients, requires development of tools for acute, reversible and preferably long-term silencing of selected neuronal subpopulations. One way to meet these requirements would be to re-engineer botulinum neurotoxins (BoNTs) [1,2,3]. These toxins target a variety of synapses, enter presynaptic terminals and then specifically cleave SNARE proteins to stop neurotransmitter release [4,5,6,7]. Restoration of neuronal activity takes place when toxin is degraded and the cleaved protein is replaced in the active zones of presynaptic terminals by the *de novo* synthesized SNARE protein [8]. Remarkably, the seven known serotypes of BoNTs cause blockade of different durations, with type A causing the longest silencing (4–6 months) in humans, whereas type E causes the shortest blockade (2–4 weeks) [9,10]. BoNTs became important research and medical tools due to their high potency, since reversible synaptic blockade can be achieved by injection of only few picograms of BoNTs in the desired location [11]. While it is possible to produce pure forms of BoNTs using normal *E. coli* expression systems, only a few labs are equipped to make full BoNTs due to safety reasons—not least their potential toxicity upon accidental injection or possible environmental contamination. The high cost of commercial BoNTs and these safety issues significantly hampered development of these silencing agents for the benefit of neurobiology and medicine. Clearly, new approaches which could solve the safety issues and also allow production of new functional variants of BoNTs could go a long way in meeting the demand for acute, on-demand neuronal silencing [12].

**Figure 1:**
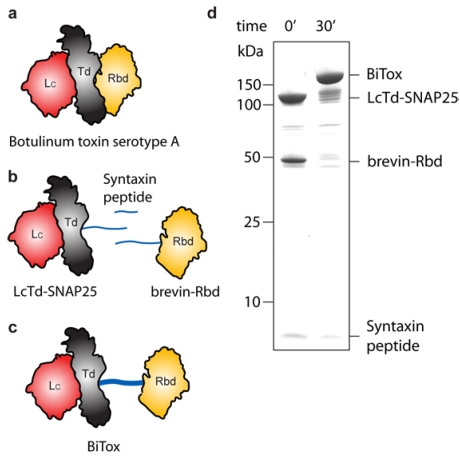
(**a**) A drawing outlining the three domains of BoNT/A (PDB 3BTA) [15]: the Light chain (Lc) in red, the Translocation domain (Td) in grey and the Receptor binding domain (Rbd) in yellow; (**b**) The three components used for assembly of BiTox are LcTd-SNAP25, brevin-Rbd and the syntaxin peptide; (**c**) The assembled BiTox consists of the same three domains of the native BoNT/A but with the SNARE stapling system between Td and Rbd domains; (**d**) Coomassie-stained SDS-PAGE gel showing the assembly of LcTd-SNAP25, brevin-Rbd and the syntaxin peptide into a single entity which represents the BiTox. The assembly reaction with all components at 5 μM concentration is complete within 30 min.

We recently reported a novel way to assemble large proteins from individual functional units [13]. We utilized the ability of three SNARE polypeptides—syntaxin, SNAP25 and synaptobrevin—to assemble in an irreversible manner into a well-defined tetra-helical complex [14]. Firstly, we fused SNAP25 to the light chain/translocation domain (LcTd) of BoNT type A (BoNT/A) and, secondly, synaptobrevin polypeptide to the receptor-binding domain (Rbd) of BoNT/A. Both fusion proteins could be expressed and purified to homogeneity. Next, we made a synthetic syntaxin peptide and demonstrated that addition of this, so-called “staple” was sufficient to assemble LcTd and Rbd into a new entity. The structural evaluation of BoNT/A (PDB 3BTA) [15] and the SNARE assembly (PDB 1SFC) [14] suggests that the re-assembled BoNT/A could be substantially longer than the native molecule (Figure 1a–c). The re-assembled BoNT/A demonstrated similar efficiency to that of native BoNT/A in cleaving SNAP25 and inhibiting neurotransmitter release in CNS neurons but exhibited a reduced potency in blocking neuromuscular junctions [13]. Here, we report our new results on the effects of the re-assembled BoNT/A (for brevity referred to as BiTox, for Binary Toxin) on CNS neurons and also provide toxicological evaluation in an animal model. Our results indicate that BiTox does not display systemic toxicity and therefore represents a promising selective CNS silencing agent.

## 2. Results

Figure 1d shows the typical assembly of BiTox from individual components visualized in a Coomassie-stained gel. Judging by the disappearance of reactants after 30 min and appearance of the high-molecular weight BiTox, it is clear that the assembly reaction is highly efficient.

**Figure 2:**
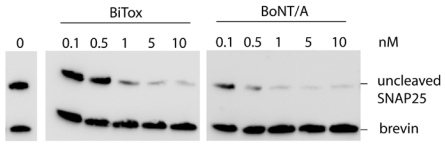
Western blot showing cleavage of SNAP25 in cultured cerebellum granule neurons by 0.1–10 nM BiTox and BoNT/A after 24 h at 37 °C. The normal immunostaining levels of SNAP25 and synaptobrevin (brevin) are shown in the untreated sample (0 nM). The SNAP25 antibody recognizes only the full-length protein but not the cleaved product.

The ability of BoNT/A to silence synaptic transmission is based on its proteolytic activity to remove nine *C*-terminal amino acids from SNAP25 [4]. Therefore, the first step in the evaluation of any engineered BoNT/A requires determination of the SNAP25 cleavage. A number of antibodies have been developed to monitor the BoNT/A-mediated SNAP25 cleavage. In the first instance, we used a *C*-terminal SNAP25 antibody that recognizes only the full-length molecule and evaluated the activity of BiTox in mouse cerebellar granule neurons. 6 days after plating, granule neurons were incubated for 24 h with the indicated dose of BiTox. After incubation with the toxin, cells were lysed and probed by western immunoblotting using the *C*-terminal SNAP25 antibody. Figure 2 shows that BiTox efficiently cleaves SNAP25 in cultured cerebellum granule neurons at 1 nM concentration.

**Figure 3:**
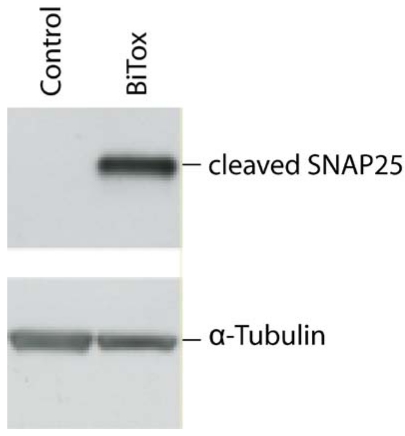
Immunoblot showing cleaved SNAP25 following intracortical injection of BiTox (4 ng). A 24 kDa band corresponding to cleaved SNAP25 is detected only in the tissue injected with BiTox, but not vehicle (Control), demonstrating that the injection of the re-assembled toxin results in the cleavage of its substrate SNAP25. α-Tubulin was used as internal standard for protein loading.

Next, we investigated whether BiTox is active when injected directly into the brain, *i.e*., not in bath conditions. Rat visual cortex was injected with either BiTox or vehicle, injection volume being 1 µL. Tissues were dissected after 2 days and subjected to western immunoblot analysis for cleaved SNAP-25. Since we anticipated that only a small area of the cortex will internalise BiTox and thus there will be an abundance of uncleaved SNAP25, we utilized an anti-peptide antibody that recognizes only the cleaved end of SNAP25 (amino acids TRIDEANQ) [16]. Figure 3 shows that 1 µL injection of 20 nM BiTox (4 ng) results in a detectable cleavage of SNAP25 within the cortex thus necessitating future behavioral and functional tests.

**Figure 4:**
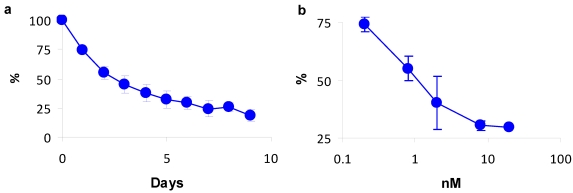
Suppression of circadian bioluminescence rhythms of suprachiasmatic nucleus (SCN) slices by BiTox. (**a**) The amplitude of the gene expression rhythms decreases dramatically following application of 0.8 nM BiTox. The luminescence peak levels were averaged and plotted as a percentage comparison with the untreated control levels (100%). Day 0 corresponds to the day of BiTox application; (**b**) Dose-dependence of BiTox-induced reduction in the luminescence peak levels 2 days following BiTox application.

To evaluate efficacy of BiTox to suppress a physiological function, we prepared organotypic slices of suprachiasmatic nucleus (SCN) from neonatal mice (10 day of age) carrying a knock-in mutation of the Period2 circadian clock gene that encodes a Per2-luciferase fusion protein [17]. Bioluminescence signals were recorded for 72 h prior to application of BiTox to confirm robust circadian oscillations. Recording continued for a further 9 days after bath addition of BiTox at indicated concentrations. Figure 4a shows that the addition of 0.8 nM BiTox halved the amplitude of the circadian oscillation within 48 h and by day 7 the peak bioluminescence was diminished to ~20% of the untreated sample, similarly to native BoNT/A [18]. Titration of BiTox activity at day 2 demonstrated IC_50_ being ~1 nM (Figure 4b).

**Figure 5:**
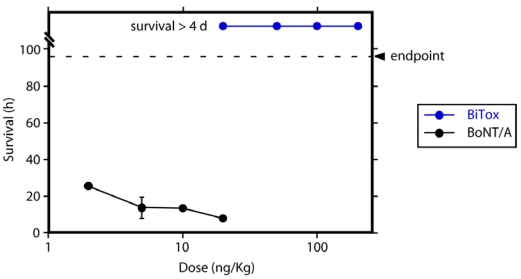
The mouse toxicity of BiTox was compared to that of native BoNT/A following intraperitoneal injection. Injections of BoNT/A at 2, 5, 10, 20 ng/kg result in lethality within 24 h, while no symptoms were detected after 96 h (the endpoint of the experiment) after i.p. injection of BiTox (20, 50, 100, 200 ng/kg doses), even at the highest concentration.

Peripheral administration of native BoNT/A causes neuromuscular blockade, which may lead to death due to paralysis of the diaphragm [19,20]. Interestingly, we previously noticed that BiTox exhibits a reduced potency at neuromuscular junctions [13] and the above intracerebral injections demonstrated no lethality, thus it was important to assess its systemic toxicity in a dose-dependent way. We evaluated the effect of intraperitoneal injections of BiTox on the musculature of the limbs and survival [21]. Following injections, the appearance of weakness of the musculature of the limbs either ipsilateral to the injection point or bilateral was monitored over 4 days. In parallel, native BoNT/A was injected. Figure 5 shows that while injections of BoNT/A were lethal even at 2 ng/kg, we have not observed obvious signs of muscle weakness in the case of BiTox within 4 days at doses up to 200 ng/kg. We conclude that BiTox does not possess the systemic toxicity evident in the case of native BoNT/A. 

## 3. Discussion and Conclusions

Our new results show that BiTox can efficiently affect CNS activities without causing neuromuscular paralysis. Such paradoxical differential action of BiTox has not been anticipated and thus requires explanation. It is highly unlikely that native BoNT/A preparations carry an unidentified component that specifically targets NMJ; indeed fully recombinant version of BoNT/A (e.g., produced by Ipsen or Merz) is a single polypeptide which has a paralytic activity comparable to the Clostridia-derived neurotoxin [22]. We favor the possibility that our re-assembled toxin retains the ability to enter central synapses but is inefficient in penetrating NMJ. The major difference between the native toxin and BiTox is that the linking mechanism results in the placement of the SNARE helical bundle (~10 nm in length, see PDB 1SFC) [14] between the receptor-binding domain (~5 nm, PDB 3BTA) and the light chain together with the translocation domain (~8 nm, PDB 3BTA) [15], thus nearly doubling the size of neurotoxin. Such an extended molecule may have a compromised ability to enter into the tight cleft of NMJs which possess hundreds of active zones extending over millimeters in length. In addition, NMJs are often covered by Schwann cell and other capping cells [23], which may also play a role in the inability of BiTox to enter the NMJ. In contrast, most CNS synapses contain a single active zone at presynaptic terminals and would be topologically more accessible even in the case of the elongated BiTox. It is also possible that BiTox has a compromised ability to translocate inside the motor nerve terminals for an unknown reason. Although the rationale of the differential action of BiTox at the NMJ and central synapses is presently unclear, the potential implications of this difference are of both biological and medical importance.

Botulinum toxin variants that specifically affect neurons and do not paralyze muscles could be invaluable when a reversible silencing of only CNS neurons is required [24,25]. For example, topical intradermal application of BoNT/A (sold under pharmaceutical names BOTOX, Dysport and Xeomin) has recently been used to alleviate pain conditions [26] and was approved for migraine treatment both in the USA and the UK. It is believed that the anti-nociceptive action of topical BoNT/A is due to the blockade of peripheral terminals of primary afferents [26]. However, because BoNT/A also causes local neuromuscular paralysis, it may be advantageous to have an improved version of the toxin which can only act on sensory terminals and not on NMJs. Another potential use of botulinum neurotoxins lies in the treatment of epilepsy [25]. Injections of botulinum neurotoxin into defined areas of the brain result in a long-term reversible silencing of the epileptic focus in animal models [27,28]. Clearly, in such CNS disorders the NMJ paralyzing ability of BoNT/A is not only redundant but also could be potentially dangerous if the injected native toxin enters the systemic circulation.

It is worth noting that the safety issues surrounding native BoNT/A significantly hamper development of this neurotoxin for the benefit of neurobiology and medicine. Clearly, new approaches that could allow development of safer versions of BoNT/A would go a long way towards meeting the need for acute, on-demand neuronal silencing. The assembly of functional BiTox from two independent and non-toxic parts, which have to be stapled together using a synthetic peptide, provides several safety levels against possible abuse. Our current results now demonstrate that the re-assembled neurotoxin possesses novel functional characteristics and can be used for silencing CNS neurons without fear of causing generalized paralysis. 

## 4. Experimental Section

### 4.1. Plasmids, Proteins Purification and Assembly

Plasmids for the expression of LcTd-SNAP25 and brevin-Rbd were previously described [13]. Briefly, all proteins were expressed in BL21 strain of E. coli as GST *C*-terminal fusions cleavable by thrombin. Proteins fused to GST were purified on glutathione Sepharose beads (GE Healthcare) and eluted from beads in 20 mM Hepes, pH 7.3, 100 mM NaCl (Buffer A) using thrombin. Further purification has been achieved by gel filtration using a Superdex 200 10/200 GL column (GE Healthcare). The BiTox was assembled by mixing the SNARE-tagged proteins with the syntaxin peptide for 30 min at 20 °C, each component at 5 μM concentration. To visualize SNARE assemblies, SDS-PAGE was performed at 4 °C, and the gels were stained with Coomassie blue.

### 4.2. Cerebellum Granule Neurons and Immunoblot

Rat cerebellar granule neurons (CGNs) were prepared from 6 days neonatal rats as previously described [29]. Briefly, fresh cerebella were disrupted mechanically in presence of trypsin and DNase I and the cells plated onto 24 well culture plates functionalized with poly-L-lysine (10 μg/mL). Cultures were maintained at 37 °C, 5% CO_2_, 95% humidity in BME supplemented with 10% fetal bovine serum, 25 mM KCl, 2 mM glutamine an 50 μg/mL gentamycin. To arrest growth of non-neuronal cells, cytosine arabinoside (10 μM) was added to the medium 18–24 h after plating. 6 days after plating, CGNs were incubated for 24 h at 37 °C with the indicated dose of either BiTox or BoNT/A (Figure 2) diluted in BME supplemented with 10% FBS and 25 mM KCl. After incubation with the toxin, cells were directly lysed in the wells with Laemmli buffer containing protease inhibitors (complete Mini EDTA-free, Roche) and separated by SDS-PAGE electrophoresis for Western immunoblotting. Primary antibodies against SNAP25 and synaptobrevin were incubated overnight at 4 °C, washed three times with PBS-Tween, and incubated with secondary antibodies conjugated to horse radish peroxidase. After three washes with PBS-Tween and one with PBS, visualization was carried out using enhanced chemiluminescence kit (ECL, GE Healthcare).

### 4.3. Western Immunoblotting of Rat Cortex Following BiTox Injection

Rats received 4 ng BiTox injection into the visual cortex (1 μL of 20 nM). Visual cortices were dissected two days following neurotoxins injection. Proteins were extracted with lysis buffer (1% Triton X-100, 10% glycerol, 20 mM Tris-HCl, pH 7.5, 150 mM NaCl, 10 mM EDTA, 0.1 mM Na_3_VO_4_, 1 μg/mL leupeptin, 1 μg/mL aprotinin, and 1mM PMSF), and the total concentration of the samples was assessed with a protein assay kit (Bio-Rad) using a bovine serum albumin-based standard curve. Immunoblotting was performed as described previously [30,31]. The antibody against BoNT/A-cleaved SNAP-25 was characterized in [31]. Protein extracts were separated by electrophoresis and blotted, and filters were incubated with primary antibodies overnight at 4 °C (anti-cleaved SNAP-25, 1:500 dilution). Blots were then reacted with HRP-conjugated secondary antibodies (Jackson ImmunoResearch) and developed by ECL (GE Healthcare). Filters were also probed with anti-α-tubulin monoclonal antibody (1:10,000 dilution; Sigma), which serves as an internal standard for protein quantification.

### 4.4. Bioluminescent Circzadian Recordings

Organotypic slices of suprachiasmatic nucleus (SCN) were made from neonatal mice (10 days of age) carrying a knock-in mutation of the Period2 circadian clock gene that encodes a Per2-luciferase fusion protein [17]. Per2-driven bioluminescence was recorded using photomultiplier tubes (Hamamatsu) as described elsewhere [32]. After 72 h to confirm robust circadian oscillations, slices were bath treated with either vehicle or BiTox at the concentrations given in Figure 3 (*n* = 3 per group). Recording continued for a further 9 days. The amplitude and precision of the gene expression rhythms were calculated using BRASS software (courtesy of A. Millar, University of Edinburgh, Edinburgh, United Kingdom).

### 4.5. Toxicity Assessment

Adult C57 BL6 mice (60–90 days of age; average 24 g) were injected intraperitoneally with the indicated doses (two mice/dose) of BiTox and native BoNT/A diluted 0.1% mouse serum albumin in PBS (injection volume 140–210 μL). Animals were transferred back in the cage and monitored every 6 h for 4 days. The endpoint of the experiment was either the appearance of weakness of the musculature of the limbs ipsilateral to the injection point or bilateral. Surviving animals were sacrificed after 4 days. All experiments were carried out under license from the UK Home Office in accordance with the Animals (Scientific Procedures) Act 1986 and following approval from the Cancer Research UK Ethical Review Committee.
